# Tai Chi Improves Brain Functional Connectivity and Plasma Lysophosphatidylcholines in Postmenopausal Women With Knee Osteoarthritis: An Exploratory Pilot Study

**DOI:** 10.3389/fmed.2021.775344

**Published:** 2022-01-03

**Authors:** Chwan-Li Shen, Bruce A. Watkins, Chanaka Kahathuduwa, Ming-Chien Chyu, Masoud Zabet-Moghaddam, Moamen M. Elmassry, Hui-Ying Luk, Jean-Michel Brismée, Ami Knox, Jaehoon Lee, Mimi Zumwalt, Rui Wang, Tor D. Wager, Volker Neugebauer

**Affiliations:** ^1^Department of Pathology, Texas Tech University Health Sciences Center, Lubbock, TX, United States; ^2^Center of Excellence for Integrative Health, Texas Tech University Health Sciences Center, Lubbock, TX, United States; ^3^Center of Excellence for Translational Neuroscience and Therapeutics, Texas Tech University Health Sciences Center, Lubbock, TX, United States; ^4^Department of Nutrition, University of California, Davis, Davis, CA, United States; ^5^Department of Laboratory Sciences and Primary Care, Texas Tech University Health Sciences Center, Lubbock, TX, United States; ^6^Department of Psychiatry, Texas Tech University Health Sciences Center, Lubbock, TX, United States; ^7^Department of Medical Engineering, Texas Tech University, Lubbock, TX, United States; ^8^Center for Biotechnology and Genomics, Texas Tech University, Lubbock, TX, United States; ^9^Department of Biological Sciences, Texas Tech University, Lubbock, TX, United States; ^10^Department of Kinesiology and Sport Management, Texas Tech University, Lubbock, TX, United States; ^11^Department of Rehabilitation Sciences, Texas Tech University Health Sciences Center, Lubbock, TX, United States; ^12^Clinical Research Institute, Texas Tech University Health Sciences Center, Lubbock, TX, United States; ^13^Department of Educational Psychology and Leadership, Texas Tech University, Lubbock, TX, United States; ^14^Department of Orthopedic Surgery, Texas Tech University Health Sciences Center, Lubbock, TX, United States; ^15^Department of Psychological and Brain Sciences, Dartmouth College, Hanover, NH, United States; ^16^Department of Pharmacology and Neuroscience, Texas Tech University Health Sciences Center, Lubbock, TX, United States; ^17^Garrison Institute on Aging, Texas Tech University Health Sciences Center, Lubbock, TX, United States

**Keywords:** mind-body exercise, neuroimaging, pain, metabolomics, WOMAC

## Abstract

**Objective:** A pre/post pilot study was designed to investigate neurobiological mechanisms and plasma metabolites in an 8-week Tai-Chi (TC) group intervention in subjects with knee osteoarthritis.

**Methods:** Twelve postmenopausal women underwent Tai-Chi group exercise for 8 weeks (60 min/session, three times/week). Outcomes were measured before and after Tai Chi intervention including pain intensity (VAS), Brief Pain Inventory (BPI), Western Ontario and McMaster Universities Osteoarthritis Index (WOMAC), plasma metabolites (amino acids and lipids), as well as resting-state functional magnetic resonance imaging (rs-fMRI, 10 min, eyes open), diffusion tensor imaging (DTI, 12 min), and structural MRI (4.5 min) in a subgroup. Clinical data was analyzed using paired *t*-tests; plasma metabolites were analyzed using Wilcoxon signed-rank tests; and rs-fMRI data were analyzed using seed-based correlations of the left and right amygdala in a two-level mixed-effects model (FSL software). Correlations between amygdala-medial prefrontal cortex (mPFC) connectivity and corresponding changes in clinical outcomes were examined. DTI connectivity of each amygdala was modeled using a Bayesian approach and probabilistic tractography. The associations between neurobiological effects and pain/physical function were examined.

**Results:** Significant pre/post changes were observed with reduced knee pain (VAS with most pain: *p* = 0.018; WOMAC-pain: *p* = 0.021; BPI with worst level: *p* = 0.018) and stiffness (WOMAC-stiffness, *p* = 0.020), that likely contributed to improved physical function (WOMAC-physical function: *p* = 0.018) with TC. Moderate to large effect sizes pre/post increase in rs-fMRI connectivity were observed between bilateral mPFC and the amygdala seed regions (i.e., left: *d* = 0.988, *p* = 0.355; right: *d* = 0.600, *p* = 0.282). Increased DTI connectivity was observed between bilateral mPFC and left amygdala (*d* = 0.720, *p* = 0.156). There were moderate-high correlations (*r* = 0.28–0.60) between TC-associated pre-post changes in amygdala-mPFC functional connectivity and pain/physical function improvement. Significantly higher levels of lysophosphatidylcholines were observed after TC but lower levels of some essential amino acids. Amino acid levels (alanine, lysine, and methionine) were lower after 8 weeks of TC and many of the lipid metabolites were higher after TC. Further, plasma non-HDL cholesterol levels were lower after TC.

**Conclusion:** This pilot study showed moderate to large effect sizes, suggesting an important role that cortico-amygdala interactions related to TC have on pain and physical function in subjects with knee osteoarthritis pain. Metabolite analyses revealed a metabolic shift of higher lyso-lipids and lower amino acids that might suggest greater fatty acid catabolism, protein turnover and changes in lipid redistribution in response to TC exercise. The results also support therapeutic strategies aimed at strengthening functional and structural connectivity between the mPFC and the amygdala. Controlled clinical trials are warranted to confirm these observed preliminary effects.

## Introduction

Knee osteoarthritis (OA), a progressive joint disease characterized by joint degeneration and inflammation (1, 2), is one of the five leading causes of disability (3). Increasing evidence shows that more patients with knee OA pain consider complementary and alternative medicine (4) to mitigate pain and improve physical function (5). Among different forms of complementary alternative medicine (CAM), Tai Chi (TC), a mind-body moderate exercise, has shown to reduce knee OA pain, and improve physical function (6–10). These prior trials suggested that TC could provide a practical exercise regimen to manage knee OA. However, TC's mechanisms of action regarding improvement of one's clinical condition and its functional outcomes in individuals with knee OA are poorly understood.

Chronic pain, such as knee OA, is associated with alterations in brain functions that modulate pain. Animal studies have shown that chronic pain is associated with increased activity of the amygdala, a brain region involved in modulating pain, emotional states, and addictive behaviors. Specifically, activity of basolateral (BLA) and central (CeA) nuclei of the amygdala increases in chronic pain (e.g., knee OA) (11, 12). Conversely, deactivation of the medial prefrontal cortex (mPFC), a brain region involved in regulating executive control, has been shown in chronic pain conditions, possibly due to enhanced feed forward inhibition by the amygdala (13). Functional magnetic resonance imaging (fMRI) and diffusion tensor imaging (DTI) are non-invasive methods that rely on magnetic resonance imaging technology. fMRI and DTI have been used to examine the effects of conditions such as chronic pain in the human brain (14–17). fMRI relies on temporal changes in oxygenated hemoglobin levels between regions of the brain to estimate the changes in functional activity between brain regions. Accordingly, temporal correlations of fMRI reactivity between brain regions serve as surrogates of functional connectivity between brain regions of interest. DTI utilizes the net rate of diffusion of water in a given direction to estimate structural (i.e., white matter) connectivity between brain regions. Taken together, resting state fMRI (rs-fMRI) and DTI allows examination of functional and structural connectivity (or neural interactions linking brain regions) between brain regions of interest in patients suffering from chronic pain.

fMRI has been used to study pain sensitization in knee OA patients (14–17). Some recent studies that specifically examined the functional reactivity of the amygdala in chronic pain using rs-fMRI have concluded that the resting state functional reactivity as well as functional connectivity of the amygdala seems to be enhanced in chronic pain conditions (18, 19). More importantly, structural connectivity in a circuit that includes the amygdala, mPFC, and the nucleus accumbens was found to be mediating the association between negative effects and the transition to chronic pain (18). This line of evidence for effects of chronic pain on functional and structural connectivity between the amygdala and mPFC corroborate results from animal experiments (11, 20–23). As such, it is logical to speculate that chronic pain disrupts the functional and structural connectivity between the amygdala and mPFC.

Adoption of metabolomics analysis to the study of OA has helped elucidate metabolic pathways and specific metabolite markers to advance the understanding of OA-caused metabolic shifts associated with systemic inflammation and oxidative stress (24–27). For instance, Zhang et al. found that compared to the non-OA subjects, the OA subjects had significantly higher concentration of acylcarnitines, but lower concentration of free carnitine in the synovial fluid, suggesting alterations in mitochondrial fatty acid oxidation in the OA status (28). Mickiewicz et al. corroborated with Zhang's study by identifying 11 metabolites in urine samples of OA subjects, but not in non-OA subjects, and authors concluded that these metabolites were mostly related to energy metabolism (29). In a case-control study, Abdelrazig et al. reported that compared to non-OA controls, knee OA subjects had altered urinary metabolic profiles that associated with perturbed activity of the TCA cycle, pyruvate, and amino acid metabolism which linked to inflammation, oxidative stress, and collagen destruction (27). Authors also showed that 2-keto-glutaramic acid level was at least 8-fold greater in the inflammatory OA patients than that in the non-OA control, suggesting a possible perturbation in glutamine metabolism related to OA progression (27).

The metabolic pathways that affect amino acid metabolism and phospholipid metabolism have been reported to associate with OA progression (24). Tootsi et al. reported that OA patients had higher levels of arginine, asparagine, leucine, serine, asymmetric dimethylarginine, phenylalanine, and spermidine, and lower levels of serotonin and spermine/spermidine ratio in serum (26). The decreased spermine/spermidine ratio is indicative of excessive oxidative stress in OA patients (26). Moreover, Tootsi et al. reported the conversion pathway of phosphatidylchoine (PC) to lysophosphatidylcholine (LPC) is overactivated in OA patients, suggesting OA patients display greater level of systemic inflammation (26). However, the effects of TC on the levels of essential and gluconeogenic amino acids, and lipid metabolites in patients with knee OA are unknown.

TC is reported to increase overall structural connectivity (i.e., white matter connectivity between two or more considered brain regions) as measured by brain DTI in elderly women (30). TC also improved rs-fMRI functional connectivity between the mPFC and the hippocampus in older adults (31). Thus, TC seems to increase both functional and structural connectivity of mPFC. However, the only rs-fMRI neuroimaging study that examined TC effects on brain connectivity in patients with knee OA, or any chronic pain condition, focused on the periaqueductal gray matter and ventral tegmental area. While this study demonstrated that exposure to 12-weeks of TC exercise reduced connectivity between ventral tegmental area and mPFC (16), the effects of TC on amygdala-mPFC interactions, which form a critical node for the processing of emotional and cognitive aspects of pain, has never been addressed. Therefore, we hypothesized that mind-body interventions such as TC may alleviate chronic pain by restoring connectivity in cortico-limbic circuits involving mPFC and the amygdala, because the mPFC as a center of the cognitive control network can modulate pain (32–34). Moreover, mind-body interventions can modulate connectivity in brain circuits centered on the mPFC (35), although pain modulation by mindfulness has been shown to occur independently of prefrontal cortical changes (36). Therefore, the objectives of this pilot study were to explore if after 8-weeks of TC exercise (i) resting state functional (i.e., rs-fMRI) and structural (i.e., DTI) connectivity between each amygdala and mPFC would increase among subjects with knee OA as compared to baseline, (ii) would modify plasma amino acid and lipid metabolites, and (iii) improve TC-associated pre-post changes between amygdala-mPFC functional connectivity and pain/physical function as well as between structural connectivity and pain/physical function. We hypothesized that 8-weeks of TC exercise would increase the amygdala-mPFC rs-fMRI and DTI connectivity of subjects with knee OA. Furthermore, TC would lead to changes in blood lipids, plasma metabolites, and lipid mediators of inflammation to improve well-being. Consistent with our hypothesis, we propose that TC changes in amygdala-mPFC rs-fMRI and DTI connectivity would correlate with reduced pain/stiffness and improved physical function.

## Materials and Methods

### Study Design

The present trial was based on a single group pre-test and post-test design to examine the effects of an 8-week TC exercise intervention on pain, physical function, stiffness, brain functional connectivity, and plasma metabolites including amino acids and lipids. Blood samples were collected at baseline and after 8 week of TC intervention. Plasma samples were stored at −80°C for later metabolites analysis. This study was approved by the Institutional Review Board at the Texas Tech University Health Sciences Center (ClinicalTrials.gov Identifier: NCT04046003).

### Recruitment of Subjects

Postmenopausal women >50 years of age with knee pain were recruited from clinics and community centers by flyers and advertisements through newspaper. The prevalence of OA increases rapidly with age, beginning at about age 40–50 years in women, but less so in men. The hormone deficiency increases the risk of OA severity in postmenopausal women. Women older than 50 have a higher prevalence and are also more likely to report joint symptoms for the same level of radiographic severity in knee OA (37). Thus, in this study, the postmenopausal women were our target study population. If women reported bilateral symptomatic knees, only the most symptomatic knee was included in the study. Written informed consent was obtained from all subjects. Participants were selected based on the following including and excluding criteria. Inclusion criteria: (1) Postmenopausal women, (2) WOMAC pain score with at least two items out of five items are rated as moderate, severe, or extreme, respectively, (3) English literacy, (4) Able to undergo an MRI scan for subjects having MRIs, (5) current pain in the knee, and (6) medical diagnosis of knee OA or knee(s) exhibited symptoms based on American College of Rheumatology clinical classification criteria for OA (38). Exclusive criteria: (1) Prior experience with mind-body practice (e.g., TC, Qi Gong, yoga, or acupuncture) or physical therapy programs for knee OA within the past 3 months, (2) Severe medical limitations (i.e., dementia, symptomatic heart or vascular disease, or recent stroke) precluding full participation, (3) Medical/neurological or other systemic diseases affecting the musculoskeletal systems (i.e., polio/Parkinson's/multiple sclerosis, rheumatoid arthritis, uncontrolled gout, etc. in addition to cerebral vascular accident or stroke) and diabetes with peripheral neuropathy affecting their sensory/balance, (4) Intra-articular steroid injection or reconstructive surgery on most severely affected knee in the past 3 months, (5) Intra-articular hyaluronic acid injections on most severely affected knee in the past 6 months, and (6) Inability to walk without an assistive device.

### Tai Chi Intervention and Compliance

The 24-form Yang style TC was employed in this study. The 24-form Yang style TC is one of the most widely practiced TC styles worldwide and one of the TC styles most widely adopted in clinical studies, with all movements well-standardized and publicized (39). The 8-week 24-form group TC program included instructed TC group classes three times per week on 3 non-consecutive days, 60 min each time at Gym of Department of Kinesiology and Sport Management, Texas Tech University, Lubbock (40, 41). Compliance of TC classes was assessed by TC class attendance record for each TC session.

Tai Chi was taught by a Master TC teacher for an 8-week period. This time selection was based on our previous studies that evaluated patients with knee OA and the effects of TC on various parameters including pain, function, range of motion, and gait parameters in 6- to 12-week trials (6, 42). The TC instructor and his assistant paid close attention to each participant during every class and made sure everyone performs TC correctly within 8 weeks. Based on our previous TC studies, all participants were able to perform the 24-form Yang style TC by themselves after 1 month. We have demonstrated in a previous study that we can teach TC and show positive effects in a group of OA participants in an even shorter period - 6 weeks (6). However, participants may subjectively expect that the intervention (Tai Chi) can improve pain, and reflect this in the subjective outcome measures (i.e., surveys), partly to please the researcher. Thus, the Hawthorne effect may be a confounder to the surveys, although not for the objective measures (e.g., fMRI, blood tests).

### Measurement of Pain, Physical Function, and Stiffness

The Western Ontario and McMaster Universities Osteoarthritis Index (WOMAC) questionnaire was used with a scale from 26 (no difficulty) to 130 (extreme difficulty) indicating the level of difficulty associated with overall functional activities due to knee pain, including subscale of knee pain (35 points), stiffness (10 points), and physical function (85 points) (6). Pain was assessed using WOMAC-pain subscale (6), visual analog scale (VAS) (6), and Brief Pain Inventory (BPI)-pain scale (43). The BPI is a validated self-reported questionnaire that assesses pain severity using the Numerical Rating Scale for Pain Intensity (NRS-PI, 0–10 scale, where 0 = no pain and 10 = worst possible pain) for the conditions of worst, least, and average pain, as well as “pain right now” (6, 43). Physical function was assessed using WOMAC-physical function and WOMAC-stiffness subscales (6) as well as BPI-interference scale (43). These measures, WOMAC (44), VAS, and BPI (6, 45), are commonly used in knee OA studies with good validity, internal consistency, and reliability.

### Measurement of Plasma Lipids

Laboratory lipid panel including total cholesterol, triglycerides, high-density lipoprotein (HDL)-cholesterol, non-HDL cholesterol, low-density lipoprotein (LDL)-cholesterol, very low-density lipoprotein (VLDL)-cholesterol, and Cholesterol/HDL ratio were assessed in plasma samples taken at baseline and 8 weeks. All samples were processed and analyzed in a certified diagnostic laboratory (Covenant Laboratory, Lubbock, TX).

### Measurement of Plasma Amino Acids and Lipids Biochemicals

The levels of metabolites in the plasma were determined using the AbsoluteIDQ™ p400 HR kit (BIOCRATES Life Sciences AG, Innsbruck, Austria) according to the manufacturer's instructions at the Center for Biotechnology & Genomics, Texas Tech University, Lubbock, TX. This assay allows the identification and quantification of more than 400 endogenous metabolites including 21 amino acids, 21 biogenic amines, 55 acylcarnitines, 18 diglycerides, 42 triglycerides, 24 LPC, 172 PC, 31 sphingomyelins, ceramides, and 14 cholesteryl esters. Identification and quantification of the metabolites was done using multiple reaction monitoring according to internal standards. Samples were analyzed by Q Exactive HF mass spectrometer coupled with a Vanquish ultra-high performance liquid chromatography (UHPLC) system (Thermo Scientific, USA). The amino acids and biogenic amines were separated using UHPLC with a C18 column (Biocrates, Part 9120052121032). Analytes were separated using a total 6 min gradient from solvent A (0.2% formic acid in water, to 95% Solvent B (0.2% formic acid in acetonitrile) per sample.

For lipid analyses, acylcarnitines, monosaccharides (hexose), diglycerides, triglycerides, LPC, PC, sphingomyelins, ceramides, and cholesteryl esters were analyzed by flow injection analysis (FIA) with total analysis time of ~3.8 min per sample with specific FIA mobile phase buffer (Part 9120052121018) provided in the kit, which was diluted into LC–MS grade methanol for use with the kit per manufacturer instructions. Using electrospray ionization in positive ion mode, samples for both UHPLC and flow injection analysis were introduced directly into Q Exactive Orbitrap MS systems (Thermo Fisher Scientific, Waltham, MA, USA) operating in the full scan or parallel reaction monitoring (PRM) mode. Acquisition methods and tune parameters for all instruments were provided by Biocrates as part of the p400HR kit. The concentrations of metabolites were calculated in μM. The LC–MS data were imported into the QuanBrowser module of the Thermo Xcalibur software (Thermo Fischer Scientific) for peak integration and quantification, then imported into MetIDQ^TM^ software package (Biocrates AG).

### Measurement of Brain Connectivity

MRI scans were performed pre-and post-intervention with the intention of modeling the resting state functional connectivity (as measured using temporal correlations between the regions) as well structural connectivity (as modeled using diffusion parameters of water in the brain) between left and right amygdala and the mPFC. Each participant underwent two MRI scanning sessions scheduled pre- and post-intervention (0 and 12 weeks, respectively). All MRI scans were performed using a 3.0 T Siemens Skyra scanner with a 20-channel head coil located at Texas Tech Neuroimaging Institute, Lubbock, TX. Each scanning session included a scout MRI scan; an rs-fMRI scan (10 min) conducted while participants kept their eyes open; a DTI scan (12 min) and a T-weighted structural MRI scan (4.5 min). Rs-fMRI data were acquired using an echo planar imaging sequence with the following parameter settings: repetition time = 3,000 ms; echo time = 30 ms; flip angle = 90; field of view = 220 mm; matrix = 64 × 64; slice thickness = 3.4 mm; and 48 ascending axial slices. Slices were tilted ~30° from the anterior commissure—posterior commissure line to minimize orbitofrontal cortical signal dropout. Diffusion weighted images were acquired using a spin-echo-based EPI sequence with the following parameters: repetition time = 10,200 ms; echo time = 82 ms; field of view = 250 mm; matrix = 125 × 125; slice thickness = 2 mm; and 80 contiguous axial slices (a non-diffusion weighted image at b = 0 s/mm^2^ and 62 volumes with uniformly distributed diffusion gradient directions at b = 1,000 s/mm^2^). The structural scan was performed using the following parameters: repetition time = 2,300 ms; echo time = 2.96 ms; flip angle = 9; field of view = 256 mm; matrix = 256 × 256; slice thickness = 1.2 mm; and 176 slices.

### Data Processing and Statistical Analysis

Descriptive statistics were calculated to inspect the distributional properties of pain, physical function, stiffness, blood lipids, and plasma metabolites. In addition, parametric and non-parametric tests were performed to examine the changes between pre- and post-intervention—paired-samples *t*-test for pain, physical function, and stiffness parameters and Wilcoxon signed-rank test for metabolite parameters. Prior to analysis, normality of pain and lipid outcomes was confirmed by skewness of the distributions (−0.80 to 1.50 at pre-intervention; 0.47–2.64 at post-intervention), as well as visual inspection of the Q-Q plots. The fMRI-related outcomes were normalized by using a Gaussian kernel. For pain, lipid, and metabolite outcomes, statistical significance was determined at an alpha level adjusted for multiple comparisons (i.e., Benjamin-Hochberg adjustment), and an effect size was computed for each comparison. All analyses were conducted using R (46).

Structural, resting-state functional and diffusion weighted raw data images were converted to NIfTI format using the dcm2nii converter (47). Structural images were pre-processed using Freesurfer (autorecon1) (48, 49). Functional images were preprocessed using tools in the FMRIB Software Library (FSL; Version 6.00, Oxford, UK). The following pre-processing steps were applied: motion correction by aligning each functional volume to the center volume within each functional run with 6-degrees of freedom (DOF) sinc interpolation using FSL's MCFLIRT tool (50); skull-stripping using FSL's BET tool (51); registration to high resolution structural space by the BBR algorithm and subsequently to the standard space by 12-DOF using FSL's FLIRT tool (52); spatial smoothing using a Gaussian kernel of FWHM 8.0 mm; grand-mean intensity normalization of the entire 4D dataset by a single multiplicative factor; high-pass temporal filtering (Gaussian-weighted least-squares straight line fitting, with sigma = 50.0 s); and FILM pre-whitening (53).

Resting-state fMRI data were analyzed using seed-based correlations. For the analyses, probability masks of each of left and right amygdala regions were created (54–59) using the Harvard-Oxford subcortical structural atlas in FSL and were converted to functional space of each participant using the FNIRT tool in FSL (60). Time-course of each seed region of pre- and postintervention scan was extracted from the pre-processed data using the fslmeants command in FSL. Resting state fMRI data were analyzed using a standard two-level pipeline in FSL. In level 1 analyses, subject level correlations between each seed region's time series and rs-fMRI data of the entire brain were analyzed using the Feat tool in FSL. An autocorrelation correction was included to account for serial dependencies between samples Woolrich 2001 (53). Level 2 analyses were equivalents of paired *t*-tests comparing the pre- vs. post-intervention level 1 estimates for each seed region (i.e., left or right amygdala). Considering the exploratory nature of the study and the limited sample size, the analyses were performed using ordinary least squares (i.e., linear approach).

Pain-associated functional dissociation of amygdala and the broader mPFC have been a consistent finding in both animal and human studies, however, there are some differences in specific sub-regions within the mPFC that showed functional dissociations. Generally, altered amygdala connectivity mainly with the infra- and pre-limbic mPFC, which are equivalent to Brodmann areas 25 and ventral 32 is reported in human subjects (13, 61–64), while human fMRI studies reported dissociated connectivity of amygdala with ventromedial PFC, including Brodmann area 25 (65) and anterior and cingulate cortical regions representing Brodmann areas dorsal 24 and 25 (64). To address potential challenges in translating the preclinical brain connectivity pattern to the human brain, we searched for clusters showing intervention-related alterations of functional connectivity with amygdala within broader target regions of mPFC (defined using a mask combing the frontal medial cortex, subcallosal cortex, paracingulate cortex and anterior cingulate cortex using Harvard-Oxford Subcortical Structural Atlas in FSL) representing Brodmann areas 32, 25, 24, and 33. Final statistical maps were thresholded using this broad mPFC mask for a z-threshold of 1.96 corresponding to a FWER corrected *p*-value of 0.05. Irrespective of the outcomes of the primary analyses, subject-level contrasts of parameter estimates of the mPFC of each level 1 analysis were extracted using the featquery tool in FSL (66). These contrasts of parameter estimates were used to calculate effect sizes. Furthermore, Spearman correlation analyses were performed using these parameter estimates between pre- vs. post-intervention changes in amygdala-mPFC functional connectivity and the corresponding changes in behavioral measures (i.e., VAS, WOMAC, and BPI) using R statistical software (4.0.2). fMRI data of one participant had to be excluded from the effect-size calculations and correlation analyses due to the contrasts of parameter estimates of both pre- and post-intervention scans being zero.

DTI data were subjected to distortion correction and brain extraction and were subsequently corrected for eddy currents and head motion using tools in the FDT Toolbox in FSL (http://fsl.fmrib.ox.ac.uk/fsl/fslwiki/FDT). The diffusion parameters of the images were estimated using a Bayesian approach (i.e., BEDPOSTX) in FSL (67, 68). Waypoint network connectivity mapping was performed using probabilistic tractography (i.e., PROBTRACX) in FSL to estimate the connectivity between each amygdala and the mPFC which defined using Harvard Oxford Cortical and Subcortical Structural Atlases in FSL (69). While not ideal given the likelihood of regression of the post-intervention outcomes toward the mean (70), considering the pilot nature of the design and the limited sample size, pre-post comparisons of the extracted total structural connectivity were performed using Wilcoxon signed-rank tests in R statistical software (4.0.2) and the corresponding effect-sizes were calculated. Moreover, Spearman correlation analyses were performed between pre- vs. post-intervention changes in amygdala-mPFC functional connectivity and the corresponding changes in behavioral measures (i.e., VAS, WOMAC, and BPI) as well as multiple biochemical markers.

## Results

### Participants

A total of 42 participants were prescreened. Among them, 17 participants met the criteria and enrolled into the study. Five participants dropped from the study due to conflict of time (*n* = 1), loss of interest with low compliance (<15%, *n* = 3), and steroid injection in knee during the study (*n* = 1). A total of 12 participants completed the 8-week study. All subjects were instructed to maintain their pre-existing physical activity, dietary habits, and medications, if any, throughout the study. A subgroup of participants (*n* = 7) received fMRI scanning for brain functional connectivity. Throughout the study, the compliance rate for TC classes was 93%. Table 1 lists demographic characteristics and medical history of study participants.

**Table 1:** Demographic characteristics of study population.

**Variables**	**Subjects (*n* = 12)**
Age [y]	64.5 ± 6.7
Weight [kg]	82.5 ± 10.2
Height [cm]	164.3 ± 6.3
Body mass index [kg/m^2^]	30.6 ± 4.1
Regular physical activities [*n* (%)]	9 (75)
Low (walking, gardening)	4 (44.4)
Medium (bicycling, swimming)	5 (55.6)
High (aerobic, running)	0 (0)
Medical history questions [*n* (%)]	
General health rated “good”	12 (100)
Total knee replacement	2 (16.7)
History of rheumatoid arthritis or gout	1 (8.3)
Heart value/cardiac bypass surgery, stent procedure, or pacemaker	2 (16.7)
History stroke or heart attack	1 (8.3)
Low back pain and/or leg pain	2 (16.7)
Hormone or hormone-like therapy use	2 (16.7)
Blood pressure drug use	3 (25)
Thyroid hormone drug use	2 (16.7)
Pain medication use	4 (33.4)
Calcium/vitamin D use	6 (50)
History of cigarettes (current or ever-smoked)	3 (25.0)
Alcohol consumption	7 (58.3)

*All data are mean ± standard deviation unless otherwise specified*.

### Pain, Physical Function, and Stiffness

**Figure 1:**
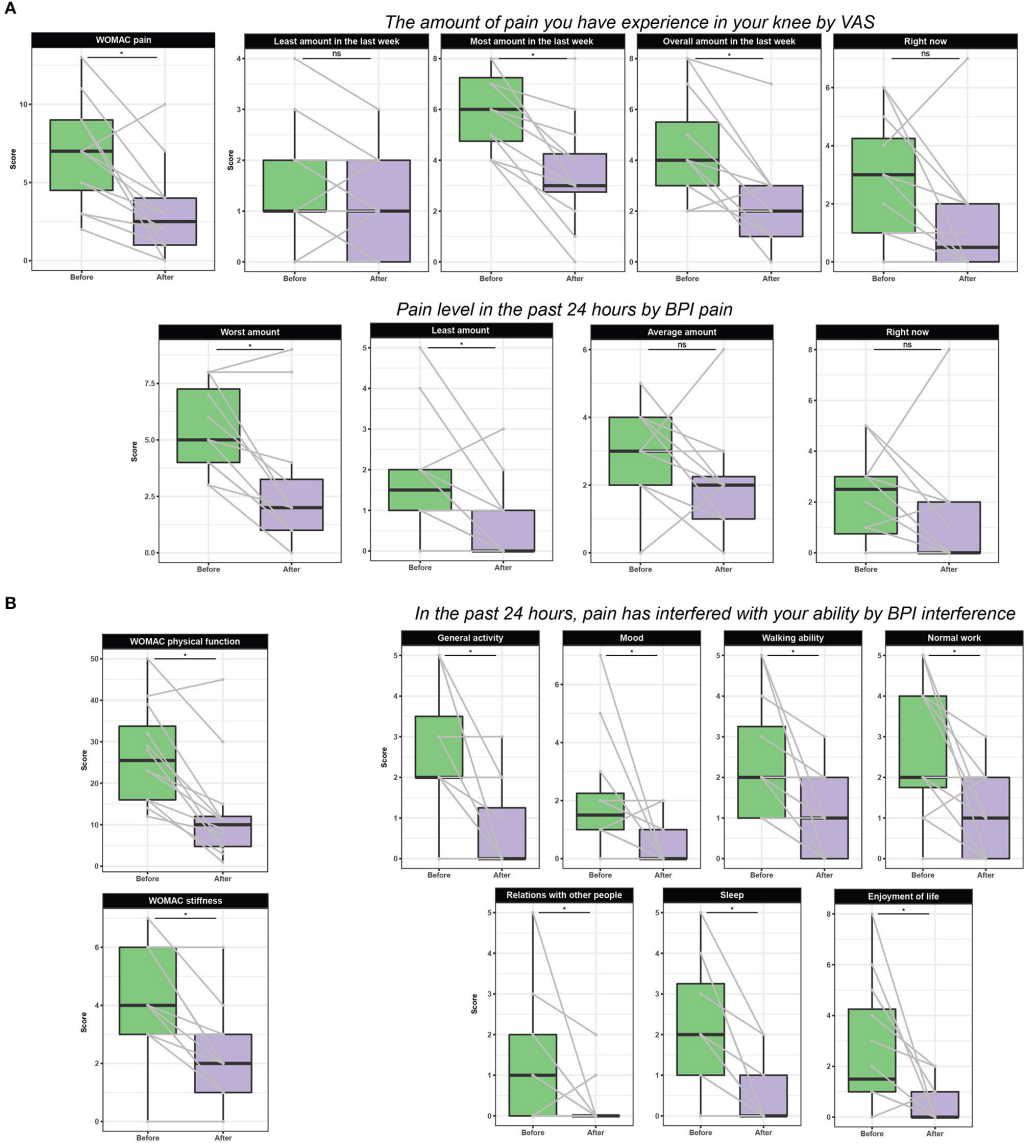
Effect of TC on pain **(A)**, physical function, and stiffness parameters **(B)**. Data are presented as boxplots and scores from the same subject is connected with a gray line. Statistical significance of differences was determined at an alpha level adjusted for multiple comparisons (i.e., Benjamin-Hochberg adjustment). WOMAC: Pain subscale from 7 (no pain) to 35 (extreme pain); Stiffness subscale from 2 (no stiffness) to 10 (extreme stiffness); Physical function subscale from 17 (no difficulties with activity of daily living) to 85 (extreme difficulties with activity of daily living). Overall WOMAC scale from 26 (best possible score) to 130 (worst possible score) due to knee pain. VAS (visual analog scale) from 0 (no knee pain) to 10 (unbearable pain). BPI (Brief Pain Inventory) from 0 (no pain) to 10 (unbearable pain).

Figure 1 presents the effect of 8-week TC on pain, physical function, and stiffness. After 8-week TC exercise, subjects with knee OA had significant pain reduction in OA-affected knee as assessed by WOMAC-pain scale (*p* = 0.021), VAS with most pain (*p* = 0.018) and overall amount in the last week (*p* = 0.018) and right now (*p* = 0.068), and BPI with worst level (*p* = 0.018) and least amount (*p* = 0.033) in the past 24 h (*p* = 0.018). Compared to the baseline, after 8-week TC exercise, the subjects reported significantly reduced stiffness at OA-affected knee by 50%, as demonstrated by WOMAC-stiffness subscale (*p* = 0.020). In terms of physical function, after 8-week TC group exercise, the subjects significantly improved their physical function as shown by WOMAC-physical function subscale (*p* = 0.018), and reduced pain-caused interference with her ability, such as general activity (*p* = 0.022), mood (*p* = 0.033), walking ability (*p* = 0.018), normal work (*p* = 0.022), relations with other people (*p* = 0.047), sleep (*p* = 0.018), and enjoyment of life (*p* = 0.018).

### Plasma Lipid Panel

Table 2 lists the effect of 8-week TC on plasma lipids. TC exercise significantly led to lower non-HDL-cholesterol levels (*p* = 0.049). Changes in non-HDL cholesterol and LDL-cholesterol were also large (Cohen's *d* ≥ 0.712), but they were not statistically significant at the adjusted alpha level. Also, TC exercise had no effect on plasma triglycerides, HDL-cholesterol, VLDL-cholesterol, and cholesterol/HDL ratio (all *p* > 0.05).

**Table 2:** Effect of group TC on lipid profiles.

**Parameters**	**Before**	**After**	***p***	**Cohen's *d***
Total cholesterol (mg/dL)	229.78 ± 64.68	219.67 ± 60.10	0.201	0.85
Triglycerides (mg/dL)	151.44 ± 79.40	152.22 ± 66.05	0.931	0.03
HDL-cholesterol (mg/dL)	57.22 ± 12.52	56.00 ± 12.86	0.931	0.22
Non-HDL cholesterol (mg/dL)	172.56 ± 63.02	163.67 ± 58.69	0.049	1.16
LDL-cholesterol (mg/dL)	141.22 ± 59.40	132.56 ± 49.68	0.326	0.71
VLDL-cholesterol (mg/dL)	30.44 ± 15.82	30.78 ± 13.00	0.931	0.06
Cholesterol/HDL ratio	4.11 ± 1.28	4.03 ± 1.26	0.931	0.26

*Mean ± standard deviation*.

*Cohen's d, effect size indicating the standardized difference in means before and after; HDL, high-density lipoprotein; LDL, low-density lipoprotein; VLDL, very low-density lipoprotein*.

### Plasma Metabolite Levels

**Figure 2:**
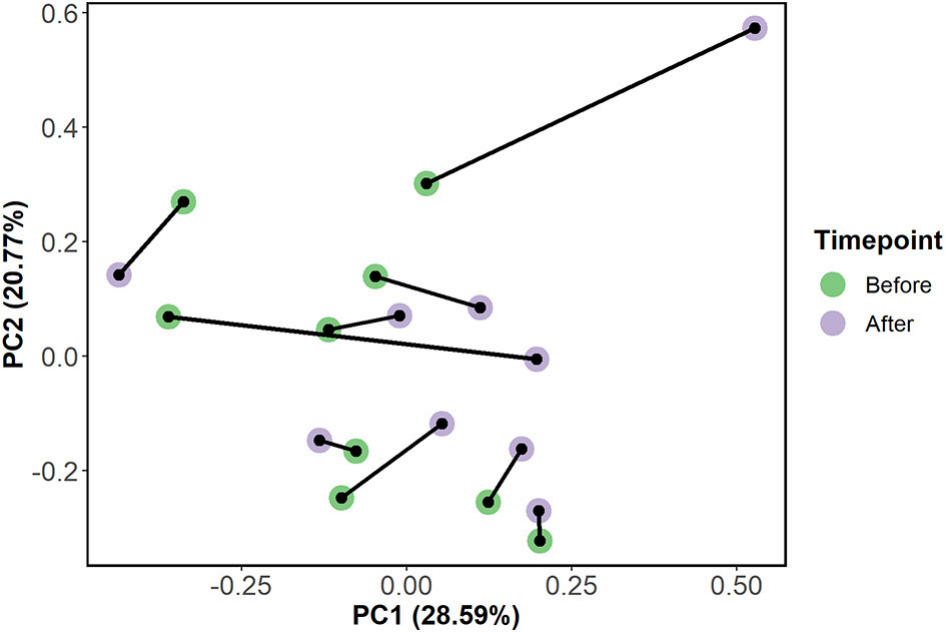
A principal component analysis (PCA) plot of the plasma metabolites concentration in the subjects (*n* = 10) before and after TC intervention. Samples are colored based on the time-point (before or after intervention) and samples from the same subject are connected by a black line.

Here, we leveraged an untargeted metabolomics approach to investigate TC effect on 336 identified plasma metabolites. These metabolites comprised several chemical classes, including acylcarnitines, diglycerides, triglycerides, LPC, PC, sphingomyelins, ceramides, and cholesteryl esters. Among all metabolites, 295 metabolites were detected in at least half of the analyzed samples, which were further used in the analysis. Principal component analysis (PCA) of the plasma metabolome profiles revealed a slight clustering of the metabolome profiles before vs. after intervention (Figure 2). Moreover, we observed a shift of the metabolome profiles along the PC1 axis, which explained 28.6% of the variance, which was indicative of changes in the concentration of the metabolites caused by the intervention.

**Figure 3:**
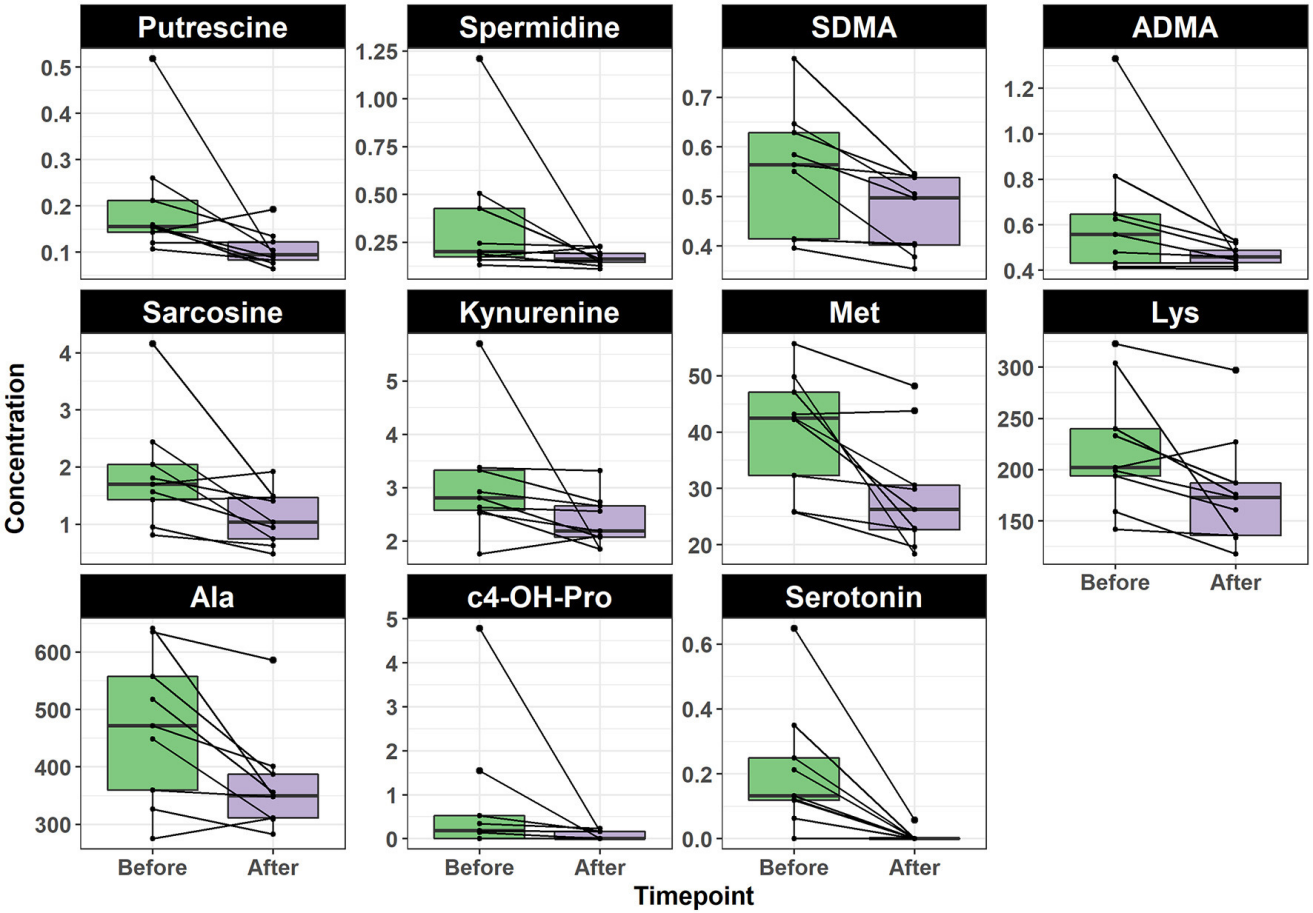
Boxplot shows the concentration of amino acids metabolites before and after TC intervention. Paired samples are linked by a line for each subject. All shown metabolites were significantly decreased after intervention (adjusted *p* < 0.05). ADMA, asymmetric dimethylarginine; Ala, alanine; c4-OH-Pro, cis-4-hydroxyproline; Lys, lysine; Met, methionine; SDMA, symmetric dimethylarginine.

**Figure 4:**
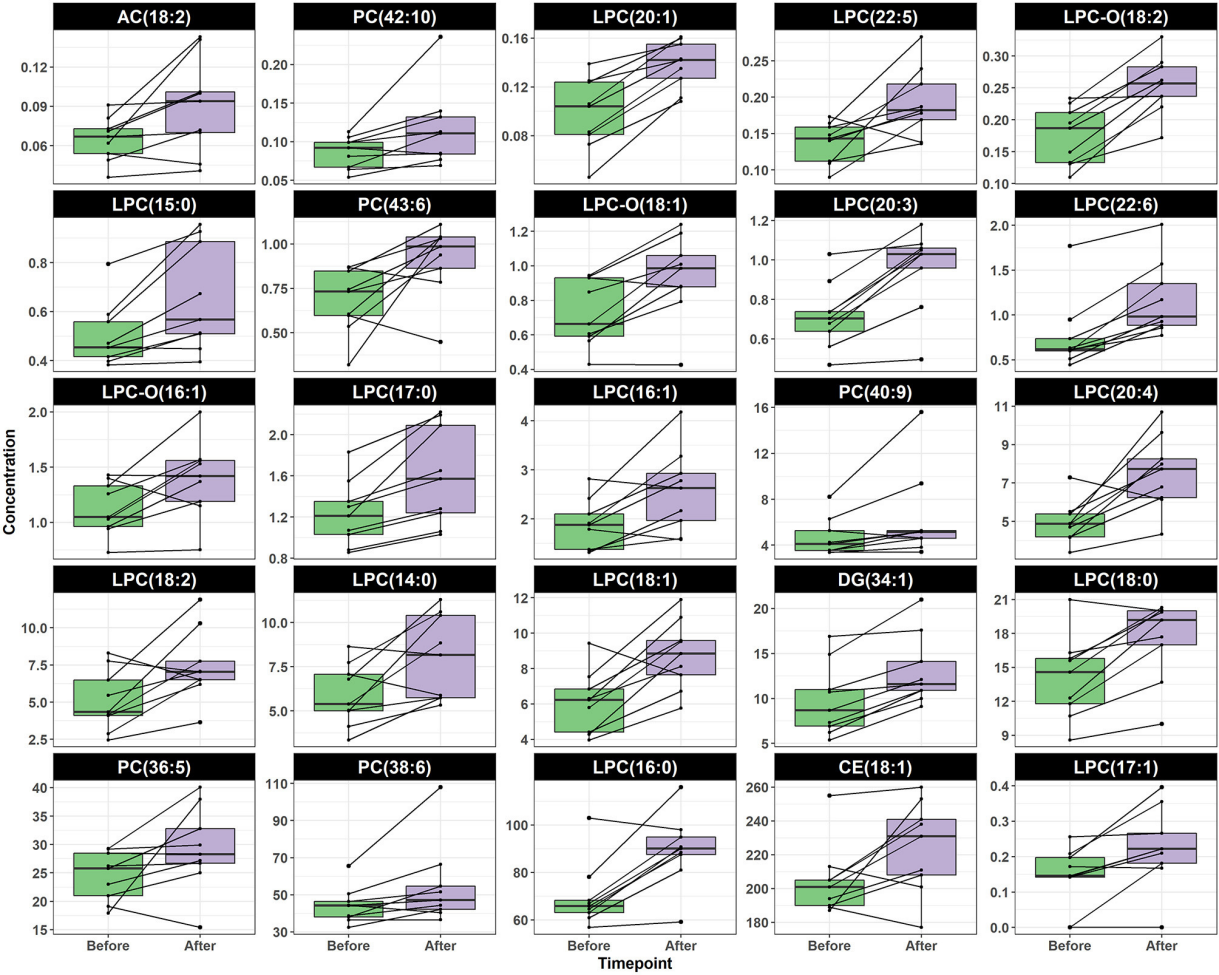
Boxplot shows the concentration of lipids metabolites before and after TC intervention. Paired samples are linked by a line for each subject. All shown metabolites were significantly increased after intervention (*p* < 0.05).

To identify the individual metabolites of which their concentration was altered by the intervention, we used the Wilcoxon signed-rank test. The change in the concentration of 36 metabolites was statistically significant (*p* < 0.05). We identified 11 and 25 metabolites that decreased and increased after intervention, respectively. Interestingly, the metabolites decreasing after intervention belonged to the chemical classes of biogenic amines and amino acids (Figure 3). The eight biogenic amines included serotonin (*p* = 0.031), *cis*-4-hydroxyproline (c4-OH-Pro) (*p* = 0.040), spermidine (*p* = 0.040), putrescine (*p* = 0.035), sarcosine (*p* = 0.035), symmetric dimethylarginine (SDMA) (*p* = 0.023), asymmetric dimethylarginine (ADMA) (*p* = 0.031), and kynurenine (*p* = 0.040). The three amino acids included methionine (*p* = 0.028) (71), alanine (*p* = 0.030), and lysine (*p* = 0.031) (Figure 3). In contrast, all metabolites that increased after intervention were lipids; 17 of which were LPC [namely, LPC(14:0), *p* = 0.035; LPC(15:0), *p* = 0.028; LPC(16:0), *p* = 0.030; LPC(16:1), *p* = 0.031; LPC(17:0), *p* = 0.030; LPC(17:1), *p* = 0.031; LPC(18:0), *p* = 0.028; LPC(18:1), *p* = 0.038; LPC(18:2), *p* = 0.040; LPC(20:1), *p* = 0.023; LPC(20:3), *p* = 0.023; LPC(20:4), *p* = 0.030; LPC(22:5), *p* = 0.031; LPC(22:6), *p* = 0.023; LPC-O(16:1), *p* = 0.040; LPC-O(18:1), *p* = 0.031; LPC-O(18:2), *p* = 0.023], five PC [namely, PC(36:5), *p* = 0.040; PC(38:6), *p* = 0.031; PC(40:9), *p* = 0.031; PC(42:10), *p* = 0.031; PC(43:6), *p* = 0.035)], one diglyceride (34:1) (*p* = 0.023), one cholesteryl ester (18:1) (*p* = 0.044), and an acylcarnitine (18:2) (*p* = 0.040) (Figure 4).

### Brain Functional and Structural Connectivity

**Figure 5:**
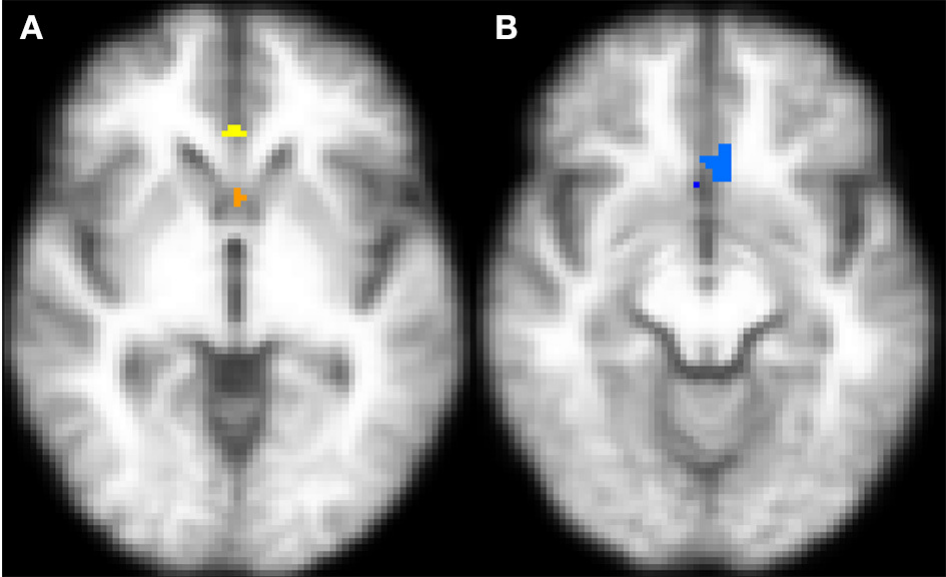
Transverse sections of the brain depicting regions within the mPFC that showed a post- vs. pre-TC intervention increase in rs-fMRI connectivity in with the **(A)** left amygdala (orange-yellow) and **(B)** right amygdala (64) after 8-week TC intervention. The functional maps have been registered on the mean of T1-weighted structural images of all participants. An increase in rs-fMRI connectivity was observed between bilateral mPFC and the left amygdala [*n* = 7, *p* = 0.355, **(A)**] and right amygdala [*n* = 7, *p* = 0.282; **(B)**] seed regions with 8 weeks of TC intervention. The images represent within-subject pre-post comparisons. After correcting to maintain family-wise error rate at 0.05, none of the observed changes remained statistically significant.

After 8 weeks of TC intervention, clusters in mPFC showed increased rs-fMRI connectivity with the left amygdala as shown in orange-yellow color (Figure 5A) and the mPFC clusters showed increased rs-fMRI connectivity with the right amygdala in blue color (Figure 5B) in the subgroup (*n* = 7) of subjects with knee OA. However, the pre-post difference was not significant (left amygdala with *d* = 0.988, *p* = 0.355, right amygdala with *d* = 0.600, *p* = 0.282). Similarly, DTI analyses revealed a pre-post increase in structural connectivity between bilateral mPFC and each of left amygdala (*p* = 0.156, *d* = 0.720) and right amygdala (*p* = 0.219, *d* = 0.528).

**Figure 6:**
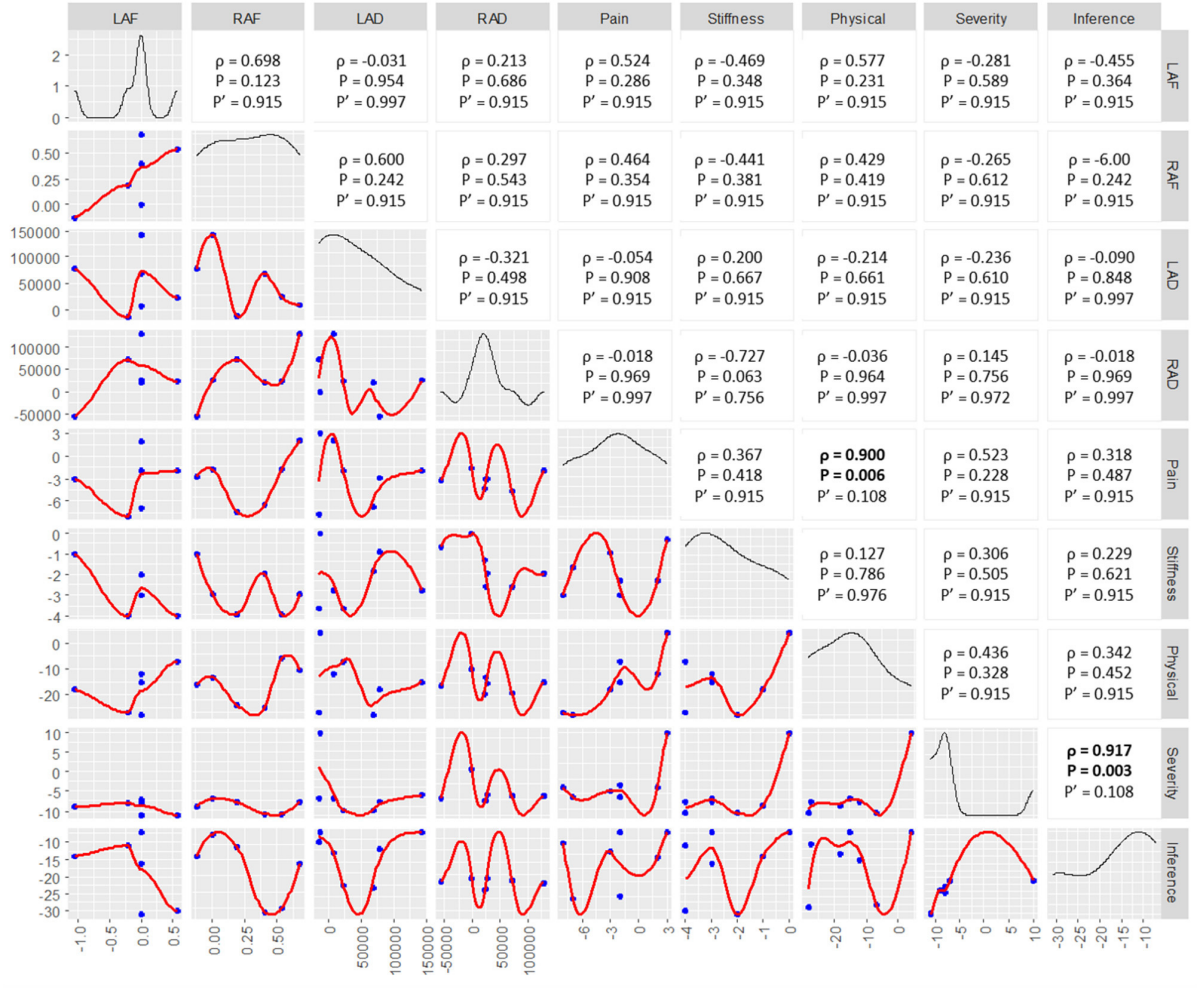
Cross correlations between post- vs. pre-TC intervention changes in amygdala-mPFC rs-fMRI / DTI connectivity and the corresponding changes in pain/physical function. The left lower half of the figure depicts univariate scatter plots with Loess regression lines. The diagonal of the figure depicts probability distribution functions of each variable. The right upper half of the figure presents the Spearman's rho (ρ) of each univariate correlation, the corresponding unadjusted *p*-value (*p*) and the *p*-value adjusted for using the Benjamini-Hochberg procedure (*p*'). LAF, L/amygdala-mPFC functional connectivity; RAF, R/amygdala-mPFC functional connectivity; LAD, L/amygdala-mPFC DTI connectivity; RAD, R/amygdala-mPFC DTI connectivity; Physical, physical function.

Figure 6 shows the correlation between post- vs. pre-TC intervention changes in fMRI connectively and physical function parameters. We found that there were moderate-strong non-linear correlations between pain and physical function parameters and the behavioral parameters, although these correlations were not significant, except for R/amygdala-mPFC DTI connectivity and stiffness with a negative correlation trend (unadjusted *p* = 0.063). For instance, there was a positive non-linear correlation between post- vs. pre-Tai Chi intervention change in physical function and L/amygdala-mPFC rs-fMRI connectivity (ρ = 0.577, unadjusted *p* = 0.231), suggesting that improvements in physical function may be associated with increased rs-fMRI connectivity (i.e., reversal of the pain-induced functional disconnect) between the L/amygdala and mPFC. Similarly, there was a negative non-linear correlation between post- vs. pre-TC intervention change in stiffness and R/amygdala-mPFC DTI connectivity (ρ = −0.727, unadjusted *p* = 0.063), suggesting that decreased stiffness with exposure to TC intervention may be associated with increased structural white matter connectivity between the R/amygdala and mPFC.

## Discussion

To our knowledge, this pilot study is the first to examine the effects of TC group intervention on both resting state functional and structural connectivity between the amygdala and mPFC. These networks involved in pain processing have been studied in humans with pain status (18, 57, 69). Connectivity between mPFC and amygdala has been identified as a predictor for chronic pain and pain vulnerability. Functional connectivity of the amygdala and mPFC has been consistently shown to increase in chronic pain, such as OA pain, indicating the importance of functional connectivity in human pain regulation (65). Resting state functional and structural (i.e., white matter) connectivity of the amygdala with mPFC has also received great attention as an important neurobiomarker of chronic pain (72) and transition to chronic pain from sub-acute states of pain (18). Thus, there is sufficient empirical support to suggest that mPFC-amygdala connectivity decreases in chronic pain states, including knee OA pain. Given that chronic pain conditions such as knee OA have been linked to decreased functional connectivity between the amygdala and mPFC, the moderate effect sizes observed in rs-fMRI or DTI connectivity in pre- and post-TC intervention we report in this pilot study reflect an increase in amygdala-mPFC connectivity after 8 weeks of TC intervention. This observed increase in structural connectivity, while not statistically significant, indicates the possibility of reversal of chronic pain associated-brain changes following TC intervention in subjects with knee OA pain. Our self-reported pain reduction results of WOMAC and BPI in OA subjects provide evidence in supporting the fMRI findings of subjects after 8 weeks of TC intervention.

Excessive oxidative stress and systemic low-grade inflammation are found to be upregulated in OA (73). In the integration of the metabolomics data along with brain functional/structural connectivity and clinical data from the present study may yield additional insights on TC and pain. To our knowledge, this study is the first to examine the effect of TC intervention on plasma amino acid and lipid metabolites along with brain functional and structural connectivity in knee OA patients.

Amino acids (AA), polyamines, and lipid biochemicals play a significant role in maintaining the balance between ROS and antioxidant systems. Chen et al. reported that in OA patients, compared to non-OA controls, the levels of 10 metabolites [alanine, arginine, creatine, c4-OH-Pro, isoleucine, leucine, lysine, tryptophan, tyrosine, and valine] were higher, whereas 12 AA metabolites [acetyl-carnitine, γ-aminobutyric acid (GABA), asparagine, citrulline, creatinine, dimethylglycine, glutamine, phenylalanine, proline, serine, and taurine] were lower (74). Among the metabolic pathways of the changed AA, the most significant involved Ala, c4-OH-Pro, arginine, aspartate, and glutamate (74). Senol et al. also reported compared to non-OA subjects, OA subjects had higher levels of alanine, isoleucine, leucine, valine, and lower levels of L-Ornithine, associated with energy metabolism (75). Alanine is connected with sclerosis in subchondral bone and may indicate greater energy consumption in OA (76). c4-OH-Pro is important to skeletal health, and is also considered as a marker of bone turnover and resorption in bone remodeling (77). In this study, the findings that TC intervention decreased plasma alanine, and c4-OH-Pro provides evidence on how TC could benefit OA patients in supporting anabolic metabolism and energy production in the no-OA control or recovery of OA.

Increased serum arginine level in OA has been associated with the nitric oxide synthase (78) function. NOS releases nitric oxide from arginine and has an endogenous metabolic inhibitor ADMA (26). Moreover, the spermine-spermidine system protects against oxidative stress by scavenging free radicals and regulating other antioxidant mechanisms (79–81). The increased circulating spermidine and lower spermine to spermidine ratio have been linked to excessive oxidative stress in OA patients. Such increased spermidine in OA patients might result by the lower activity of spermine synthase, an enzyme that converts spermidine to spermine. The accumulation of spermidine impairs lysosome function and leads to increase oxidative stress (80). Tootsi et al. reported that (1) an elevated level of ADMA in OA patients that suppresses NOS activity, results in decline utilization of arginine and (2) significantly increased levels of arginine, asparagine, leucine, serine, phenylalanine, and spermidine, whereas the ratio of spermine to spermidine was decreased in the OA patients, reflecting an excess of oxidative stress (26). In the present study, the findings that TC intervention significantly reduced arginine metabolites (i.e., SDMA and ADMA) and spermidine provide evidence of TC's anti-oxidative stress action. Our finding corroborates with the previous studies showing TC exercise reduced oxidative stress, as shown by reduced urinary 8-hydroxy-2'-deoxyguanosine (oxidative stress biomarker) (81) and serum lipoperoxides (82–85).

Serotonin is mostly known for its function as a neurotransmitter and it has been found to be involved in pain and inflammation, showing pro- and anti-nociceptive effects of serotonin through the distinct receptor (86). Seidel et al. reported serotonin mediates PGE_2_ overexpression through 5-HT2A and 5-HT3 receptor subtypes in serum-free tissue culture of macrophage-like synovial cells, suggesting the involvement of elevated serotonin in progression of OA (87). Ji et al. also reported that knockdown of 5-HT2C in the amygdala has beneficial effects on chronic pain, which is in line with the data reported here that TC exerts beneficial effects through reduction of 5-HT (88).

The kynurenine pathway of tryptophan metabolism has been implicated in the pathogenesis of inflammation, including OA (30, 88, 89). Lögters et al. demonstrated that (i) synovial kynurenine values and ratio of kynurenine/tryptophan in septic arthritis patients were significantly increased compared to patients with non-infectious inflammatory arthropathy or OA (30), and there is a significant positive correlation between kynurenine values and synovial interleukin-1β and interleukin-6 in synovial fluid, indicating that the activation of kynurenic pathway is a consequence of inflammation (89). Intriguingly, Lögters' findings would provide an explanation for TC's anti-inflammation on OA as shown by significant reduction in plasma kynurenine of OA subjects.

A number of studies have identified altered status of glycolysis and glucose metabolism glycolytic proteins in OA (90, 91). For example, Anderson et al. reported that OA patients had significantly higher levels of substrates for glycolysis and the TCA cycle, including sarcosine, glucose, mannose, pyruvate and citrate. In addition, many amino acids, which feed into glycolysis and the TCA cycle, were higher in OA, including alanine, tyrosine, glutamine, proline, histidine, asparagine, and taurine (92). In the present study, we reported TC intervention resulted in a reduction in plasma putrescine, sarcosine, and alanine of OA subjects, suggesting TC may play an important role in chondrocyte protection of OA *via* improving glucose metabolism. Future study is warranted to confirm our pilot findings.

While our study focused on brain circuits to explain beneficial effects of TC, the various plasma metabolites implicated in TC here could also act on peripheral targets such as transient receptor potential vanilloid 1 (TRPV1), which is expressed in nociceptors and has been linked to OA and OA pain in preclinical and clinical studies (90, 91). TRPV1 was discovered as a thermosensor and subsequently a nociceptor by Nobel Laureate Dr. David Julius (93). TRPV1 signaling can be activated or facilitated by a number of endogenous factors and intracellular signaling mechanisms, including extracellular protons at levels found in tissue acidosis associated with arthritis and other conditions (94), cannabinoid anandamide (95), bioactive lipids such as LPC (96), and PLA2 (97); these may be modulated in TC because we found changes in amino acids, lipids, PC to LPC levels and evidence for conversion (98).

OA patients have impaired lipid metabolism, which might be the result of increased energy requirement and decreased supply of lipids (99). Severe OA patients may have higher energy requirements (hypertrophic chondrocytes, production of inflammatory mediators); at the same time, the energy supply from lipid β-oxidation may be impaired (inadequate enzyme functioning) (99). In a case-control study, Tootsi et al. reported medium- and long-chain acylcarnitines were significantly lower in the OA subjects than the non-OA subjects, and the acylcarnitines levels were negatively associated with OA severity as well as with arterial stiffness in end-stage OA patients (99). Thus, acylcarnities might play an important role in the association between OA and cardiovascular disease (99); however, the investigators used a kit for the acylcarnitine measurements. The lower level of acylcarnitines might be caused by carnitine deficiency or by increased energy consumption in OA. One of the causes of increased energy expenditure is inflammation. In the present study, TC intervention resulted in higher plasma acylcarnitines levels determined by UHPLC, suggesting TC's potential in reducing inflammation which was reported previously (100). In contrast, others observed that 8-week TC intervention reduced non-HDL cholesterol of knee OA subjects agrees with published meta-analysis work (101). Different from published work with subjects at high risk of cardiovascular disease, our study is the first study in postmenopausal women with knee OA pain. The higher plasma acylcarnitines after TC intervention would show TC's benefit on OA patients in the aspect of cardiovascular health.

In animal OA models, Pousinis et al. suggested potential OA biomarkers to include those associated with cholesterol biosynthesis, sphingolipid metabolism, and arachidonic acid metabolism, and linoleic acid, alpha-linolenic acid, and glycerophospholipid (102). Overall cholesteryl ester (CE) (18:2), CE (20:4), and CE (22:6) levels were positively correlated with pain behavior (102). In our study, we found CE (18:1) and diglyceride (103) (34:1) elevated in OA subjects after 8 weeks of TC intervention. Interestingly, we also found that TC exercise led to higher levels of diglyceride and cholesterol ester, suggesting TC exercise could increase catabolism of lipids.

Metabolites of PC and LPC are shown to associate with the progression of OA (28, 99, 100). Tootsi et al. reported compared to non-OA group, OA group had significantly lower serum LPC acyl C14:0, PC diacyl C30:0, PC diacyl C32:2, PC diacyl C32:3, PC diacyl C34:3, PC diacyl C34:4, PC acyl-alkyl C30:0, PC acyl-alkyl C34:2 and PC acyl-alkyl C34:3, and higher levels of LPC acyl C20:4, PC diacyl C38:6, PC diacyl C40:6, and SM C20:2, suggesting the conversion pathway of PC to LPC being overactivated in OA patients with greater level of systemic inflammation (26). In the present study, we identified 25 lipid metabolites that were elevated after TC intervention. Among these 25 elevated lipid metabolites, 68 and 20% were classified as LPC and PC, respectively, with the remaining metabolites annotated as acylcarnitines (AC 18:2), diglycerides (103), and cholesteryl esters. The finding that elevation of plasma LPC and PC levels in knee of OA subjects after 8 weeks of TC intervention suggests TC's anti-inflammatory action.

In this pilot study, we did find connectivity improvements between the amygdala and mPFC with TC intervention after appropriate thresholds, but they did not reach a level of statistical significance. The lack of significance could be due to the following reasons/limitations: (i) a pre-post setting with a small sample size for fMRI assessment which may be a high likelihood of the correlation being driven by extreme outliers (28), (ii) lack of a comparison group without TC intervention which may result in the observed effects could simply be due to regression toward the mean, and (iii) due to the limited sample size, thresholding of functional images was performed using an ordinary least squares approach, which is known to inflate type I error rates (104). Thus, caution is advised in interpreting the pre-post comparisons a well as the magnitude of correlations in this preliminary, exploratory study. A future randomized control study to include a comparison group without TC intervention with adequate power is warranted to confirm the observed effects of the present study, with a focus on how TC, a mind-body exercise, might strengthen functional and structural connectivity between the mPFC and the amygdala.

## Conclusion

This study revealed moderate to large effect sizes, suggesting an important role for cortico-amygdala interactions due to TC intervention on pain and physical function in postmenopausal women with knee OA. TC intervention increases lysophosphatidylcholines and decreases some essential amino acids in plasma of postmenopausal women with knee OA.

## Data Availability Statement

The raw data supporting the conclusions of this article will be made available by the authors, without undue reservation.

## Ethics Statement

The studies involving human participants were reviewed and approved by Institutional Review Board at Texas Tech University Health Sciences Center. The patients/participants provided their written informed consent to participate in this study.

## Author Contributions

C-LS, BW, CK, M-CC, H-YL, J-MB, JL, MZ, TW, and VN: conceptualization and methodology. CK, M-CC, MZ-M, ME, H-YL, J-MB, AK, JL, and RW: data collection and analysis. C-LS, BW, CK, MZ-M, ME, and VN: writing—original draft preparation. C-LS, BW, CK, M-CC, MZ-M, ME, H-YL, J-MB, JL, TW, and VN: writing—review and editing. C-LS and VN: supervision, project administration, and funding acquisition. All authors have read and agreed to the published version of the manuscript.

## Funding

This study was supported by Center of Excellence for Translational Neuroscience and Therapeutics, Texas Tech University Health Sciences Center, Lubbock, TX (grant no: PN-CTNT 2018-12 LSVNBAW).

## Conflict of Interest

The authors declare that the research was conducted in the absence of any commercial or financial relationships that could be construed as a potential conflict of interest.

## Publisher's Note

All claims expressed in this article are solely those of the authors and do not necessarily represent those of their affiliated organizations, or those of the publisher, the editors and the reviewers. Any product that may be evaluated in this article, or claim that may be made by its manufacturer, is not guaranteed or endorsed by the publisher.
